# Supplementary material for: Isolation and characterization of a novel lytic bacteriophage Pv27 with biocontrol potential against Vibrio parahaemolyticus infections in shrimp
Source: PeerJ. 2025 May 6;13:e19421. doi: 10.7717/peerj.19421 (PMC12063606; doi:10.7717/peerj.19421)
Supplement: Supplemental Information 1 — A total of 267 ORFs which are predicted to encode for hypothetical proteins are omitted from the table. [file peerj-13-19421-s001.pdf]

Supplementary Table S1.
Putative ORFs in Vibrio phage Pv27 genome and their assigned functions.
A total of 267 ORFs which are predicted to encode for hypothetical proteins are omitted from the table.

ORF	Strand	Start	Stop	Annotation	Functional group	Best BLAST hit	
						% Identify	Organism
3	+	613	1221	deoxynucleoside monophosphate kinase	other	99.01	Vibrio phage phiKT1024
5	+	1506	2807	virion structural protein	head and packaging	100	Vibrio phage phiKT1024
6	+	2889	4337	ATP-dependent DNA ligase	DNA, RNA and nucleotide metabolism	98.96	Vibrio phage phiKT1024
8	+	4755	5306	methyltransferase	other	97.51	Vibrio phage phiKT1024
14	+	8800	9105	co-chaperonin GroES	other	100	Vibrio phage phiKT1024
18	-	11208	10480	head-tail adaptor Ad2	connector	99.17	Vibrio phage Va1
19	+	11272	11745	head closure	connector	98.73	Vibrio phage VB_VaC_TDDLMA
20	-	12433	11747	head closure Hc2	connector	99.56	Vibrio phage phiKT1024
21	+	12504	14006	portal protein	head and packaging	100	Vibrio phage phiKT1024
23	+	14205	16151	tail sheath	tail	99.69	Vibrio phage phiKT1024
24	+	16284	17864	chaperonin groEL	other	98.86	Vibrio phage phiKT1024
25	+	17960	18736	tail tube	tail	100	Vibrio phage phiKT1024
26	+	18746	19498	tail tube	tail	99.6	Vibrio phage phiKT1024
27	+	19537	20118	tail tube	tail	100	Vibrio phage phiKT1024
29	+	20396	20944	DNA end protector	DNA, RNA and nucleotide metabolism	99.45	Vibrio phage phiKT1024
31	+	21200	21898	head maturation protease	head and packaging	100	Vibrio phage phiKT1024
32	+	21960	22688	head scaffolding protein	head and packaging	99.59	Vibrio phage phiKT1024

33	+	22753	24114	major head protein	head and packaging	100	Vibrio phage phiKT1024
36	-	25378	24857	dihydrofolate reductase	DNA, RNA and nucleotide metabolism	100	Vibrio phage phiKT1024
37	-	26251	25388	thymidylate synthase	DNA, RNA and nucleotide metabolism	99.65	Vibrio phage phiKT1024
38	-	26709	26299	tail assembly chaperone	tail	100	Vibrio phage phiKT1024
40	+	27342	27992	baseplate hub	tail	100	Vibrio phage phiKT1024
41	+	27995	28684	baseplate protein	tail	98.89	Vibrio phage phiKT1024
42	+	28681	31317	endolysin	lysis	99.89	Vibrio phage phiKT1024
43	+	31329	32174	baseplate tail tube cap	tail	100	Vibrio phage phiKT1024
46	+	33813	34832	baseplate hub subunit and tail lysozyme	tail	99.41	Vibrio phage phiKT1024
48	+	35411	35767	baseplate wedge subunit	tail	100	Vibrio phage phiKT1024
49	+	35780	37771	baseplate protein	tail	99.85	Vibrio phage phiKT1024
53	-	43174	42683	tail tube	tail	100	Vibrio phage phiKT1024
55	+	43332	44162	RNA polymerase sigma factor	transcription regulation	99.64	Vibrio phage phiKT1024
58	-	45110	44571	endonuclease VII	DNA, RNA and nucleotide metabolism	100	Vibrio phage phiKT1024
62	+	46511	47845	HNH endonuclease	DNA, RNA and nucleotide metabolism	100	Vibrio phage phiKT1024
71	+	50709	51548	ABC transporter	moron, auxiliary metabolic gene and host takeover	99.64	Vibrio phage phiKT1024
82	+	55019	55885	endolysin	lysis	97.92	Vibrio phage phiKT1024

99	+	63490	63855	homing endonuclease	DNA, RNA and nucleotide metabolism	92.74	Vibrio phage phiKT1024
118	+	68921	69832	head maturation protease	head and packaging	98.68	Vibrio phage VB_VaC_TDDLMA
125	+	71733	72092	GTP-binding domain	other	100	Vibrio phage phiKT1024
134	+	74533	76395	anaerobic ribonucleoside reductase large subunit	DNA, RNA and nucleotide metabolism	99.33	Vibrio phage phiKT1024
135	+	76395	76838	anaerobic ribonucleotide reductase small subunit	DNA, RNA and nucleotide metabolism	98.64	Vibrio phage phiKT1024
145	+	80930	82042	2-aminooxy adenylosuccinate synthetase	DNA, RNA and nucleotide metabolism	97.3	Vibrio phage VB_VaC_TDDLMA
146	+	82054	82698	exonuclease	DNA, RNA and nucleotide metabolism	100	Vibrio phage phiKT1024
149	+	83311	83733	tRNA amidotransferase	DNA, RNA and nucleotide metabolism	92.14	Vibrio phage VB_VaC_TDDLMA
152	+	84329	85837	RNA-binding protein	DNA, RNA and nucleotide metabolism	99.8	Vibrio phage phiKT1024
163	+	89401	89928	metal-dependent phosphohydrolase	other	99.43	Vibrio phage phiKT1024
166	+	91506	91970	bifunctional heptose 7-phosphate kinase/heptose 1-phosphate adenyltransferase	other	99.35	Vibrio phage Va1
169	+	92433	93542	ATPase	other	100	Vibrio phage phiKT1024
172	+	95255	95566	thioredoxin domain	DNA, RNA and nucleotide metabolism	93	Vibrio phage VB_VaC_TDDLMA

174	+	95950	96408	Rz-like spanin	lysis	100	Vibrio phage phiKT1024
175	+	96395	96772	Rz-like spanin	lysis	100	Vibrio phage phiKT1024
177	+	97197	97781	head maturation protease	head and packaging	99.5	Vibrio phage phiKT1024
178	+	97913	98419	inhibitor of host transcription	transcription regulation	100	Vibrio phage phiKT1024
187	+	102013	102558	membrane protein	moron, auxiliary metabolic gene and host takeover	98.9	Vibrio phage phiKT1024
200	+	106185	106862	unknown function	unknown function	99.5	Vibrio phage Va1
204	+	108967	109899	clamp loader of DNA polymerase	DNA, RNA and nucleotide metabolism	99.68	Vibrio phage Va1
209	+	111108	112286	ATPase	other	99.23	Vibrio phage phiKT1024
210	+	112368	113987	DNA polymerase	DNA, RNA and nucleotide metabolism	98.32	Vibrio phage phiKT1024
212	+	114231	115304	DNA polymerase	DNA, RNA and nucleotide metabolism	97.76	Vibrio phage phiKT1024
219	+	117475	117858	clamp loader of DNA polymerase	DNA, RNA and nucleotide metabolism	100	Vibrio phage phiKT1024
233	+	121790	122314	RNA ligase	DNA, RNA and nucleotide metabolism	99.42	Vibrio phage phiKT1024
236	+	122844	124400	DnaB-like replicative helicase	DNA, RNA and nucleotide metabolism	99.81	Vibrio phage phiKT1024
245	-	129019	127121	terminase large subunit	head and packaging	100	Vibrio phage phiKT1024
252	+	130637	131218	phosphoesterase	other	98.96	Vibrio phage phiKT1024

257	+	132592	133488	RNA ligase and tail fiber protein attachment catalyst	tail	98.65	Vibrio phage phiKT1024
259	+	133990	134403	ATPase	other	100	Vibrio phage phiKT1024
260	+	134410	134595	Lar-like restriction alleviation protein	moron, auxiliary metabolic gene and host takeover	98.36	Vibrio phage phiKT1024
264	+	135550	136893	tRNA nucleotidyltransferase	DNA, RNA and nucleotide metabolism	99.33	Vibrio phage phiKT1024
265	+	136896	137390	hydrolase	other	98.78	Vibrio phage phiKT1024
269	+	138633	139226	DNA binding protein	DNA, RNA and nucleotide metabolism	93.91	Vibrio phage phiKT1024
286	-	149679	147787	long tail fiber protein distal subunit	tail	99.21	Vibrio phage phiKT1024
287	-	151216	149735	tail fiber protein	tail	100	Vibrio phage phiKT1024
288	-	152615	151248	tail sheath	tail	99.56	Vibrio phage phiKT1024
290	+	153011	154813	DNA topoisomerase II large subunit	DNA, RNA and nucleotide metabolism	99.17	Vibrio phage phiKT1024
295	+	156370	157710	DNA topoisomerase II	DNA, RNA and nucleotide metabolism	92.28	Vibrio phage phiKT1024
299	+	159272	160663	PhoH-like phosphate starvation-inducible	other	99.78	Vibrio phage phiKT1024
301	+	161259	161918	protease	other	100	Vibrio phage phiKT1024
305	+	163048	164010	partition protein	DNA, RNA and nucleotide metabolism	99.69	Vibrio phage Va1
314	+	166570	167274	DNA polymerase processivity factor	DNA, RNA and	99.57	Vibrio phage Va1

					nucleotide metabolism		
315	+	167421	168350	single strand DNA binding protein	DNA, RNA and nucleotide metabolism	97.09	Vibrio phage phiKT1024
316	+	168485	169552	exonuclease	DNA, RNA and nucleotide metabolism	98.59	Vibrio phage phiKT1024
318	+	169898	170896	DNA primase	DNA, RNA and nucleotide metabolism	99.7	Vibrio phage phiKT1024
323	+	172015	172698	exonuclease	DNA, RNA and nucleotide metabolism	98.24	Vibrio phage phiKT1024
328	+	174413	174832	ParB-like partition protein	DNA, RNA and nucleotide metabolism	98.56	Vibrio phage phiKT1024w
329	+	174841	175842	beta-glucosyl-HMC-alpha-glucosyltransferase	other	99.7	Vibrio phage phiKT1024
331	+	175928	178618	helicase	DNA, RNA and nucleotide metabolism	99.55	Vibrio phage phiKT1024
345	+	182661	184199	DNA helicase	DNA, RNA and nucleotide metabolism	99.22	Vibrio phage phiKT1024
352	+	186489	187295	DNA methyltransferase	other	99.63	Vibrio phage phiKT1024
353	+	187423	188565	ribonucleoside diphosphate reductase small subunit	DNA, RNA and nucleotide metabolism	99.47	Vibrio phage phiKT1024
354	+	188604	190949	ribonucleotide reductase large subunit	DNA, RNA and nucleotide metabolism	99.74	Vibrio phage phiKT1024
