# Supplementary material for: Isolation and characterization of a novel lytic bacteriophage Pv27 with biocontrol potential against Vibrio parahaemolyticus infections in shrimp
Source: PeerJ. 2025 May 6;13:e19421. doi: 10.7717/peerj.19421 (PMC12063606; doi:10.7717/peerj.19421)
Supplement: Supplemental Information 2 — The shading below each genome indicates sequence similarities between the genomes, with different colors representing the levels of similarity. [file peerj-13-19421-s002.pdf]

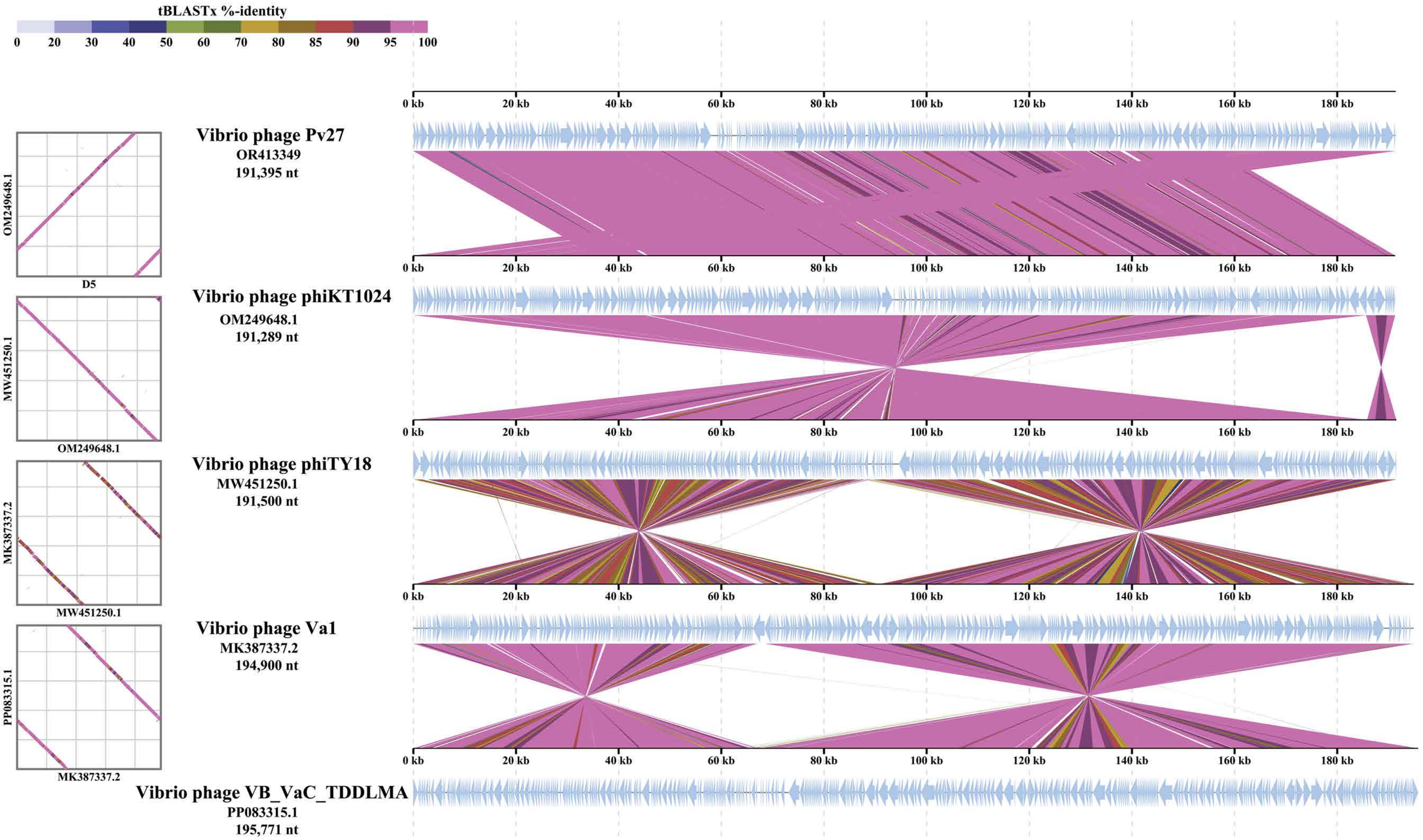

tBLASTx %-identity
0 20 30 40 50 60 70 80 85 90 95 100
0 kb 20 kb 40 kb 60 kb 80 kb 100 kb 120 kb 140 kb 160 kb 180 kb
Vibrio phage Pv27
OR413349
191,395 nt
OM249648.1
D5
Vibrio phage phiKT1024
OM249648.1
191,289 nt
MW451250.1
OM249648.1
Vibrio phage phiTY18
MW451250.1
191,500 nt
MK387337.2
MW451250.1
Vibrio phage Va1
MK387337.2
194,900 nt
PP083315.1
MK387337.2
Vibrio phage VB_VaC_TDDLMA
PP083315.1
195,771 nt
