# Supplementary figures and images for: Isolation and characterization of a novel lytic bacteriophage Pv27 with biocontrol potential against Vibrio parahaemolyticus infections in shrimp
Source: PeerJ. 2025 May 6;13:e19421. doi: 10.7717/peerj.19421 (PMC12063606; doi:10.7717/peerj.19421)

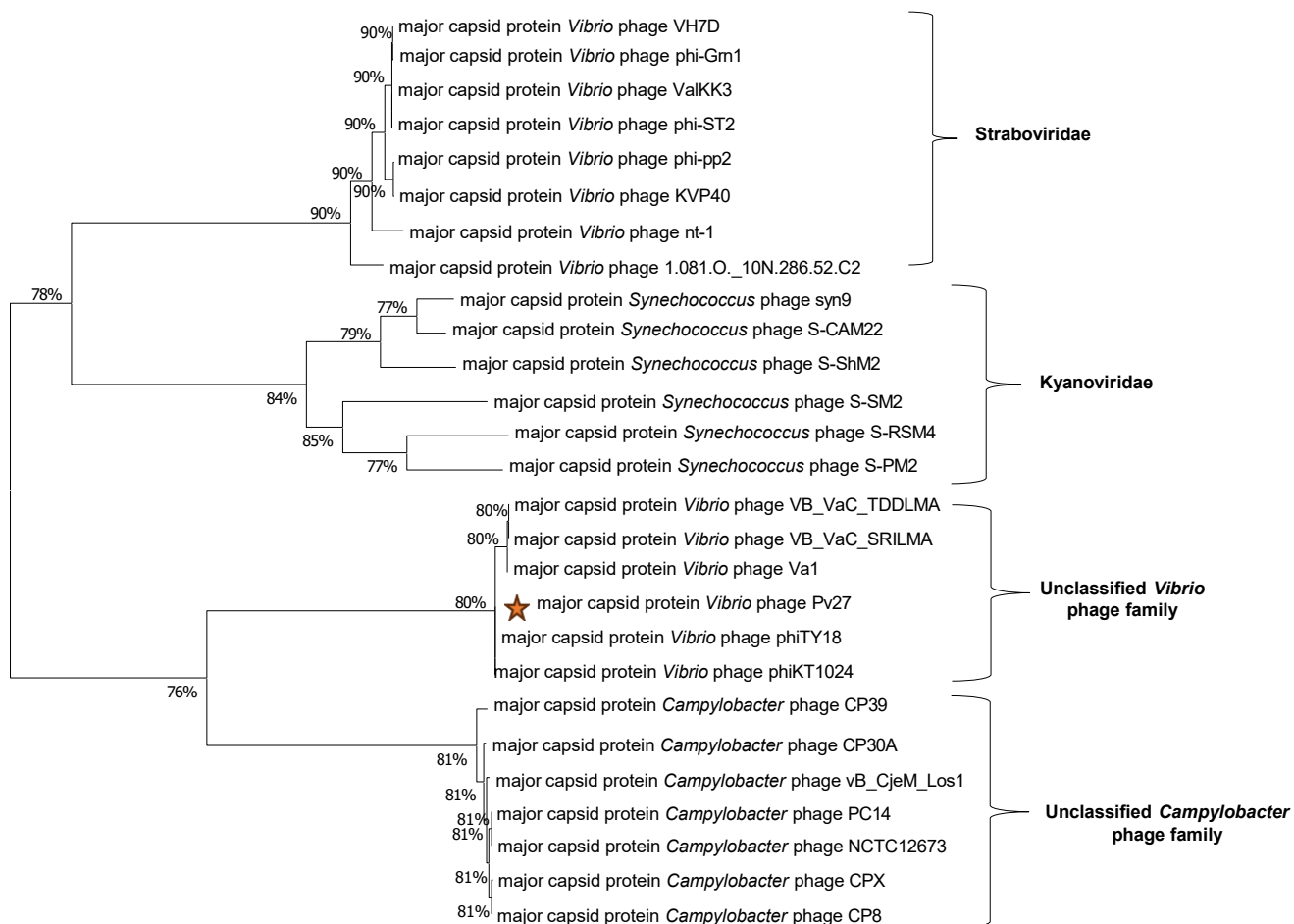

Supplement: Supplemental Information 3 — The phylogenetic tree was constructed using the Maximum Likehood with 1000 bootstrap replicates. Reference sequences were selected based on the top-hit BLAST results of phage Pv27’s major capsid amino acid sequences. [file peerj-13-19421-s003.pdf]

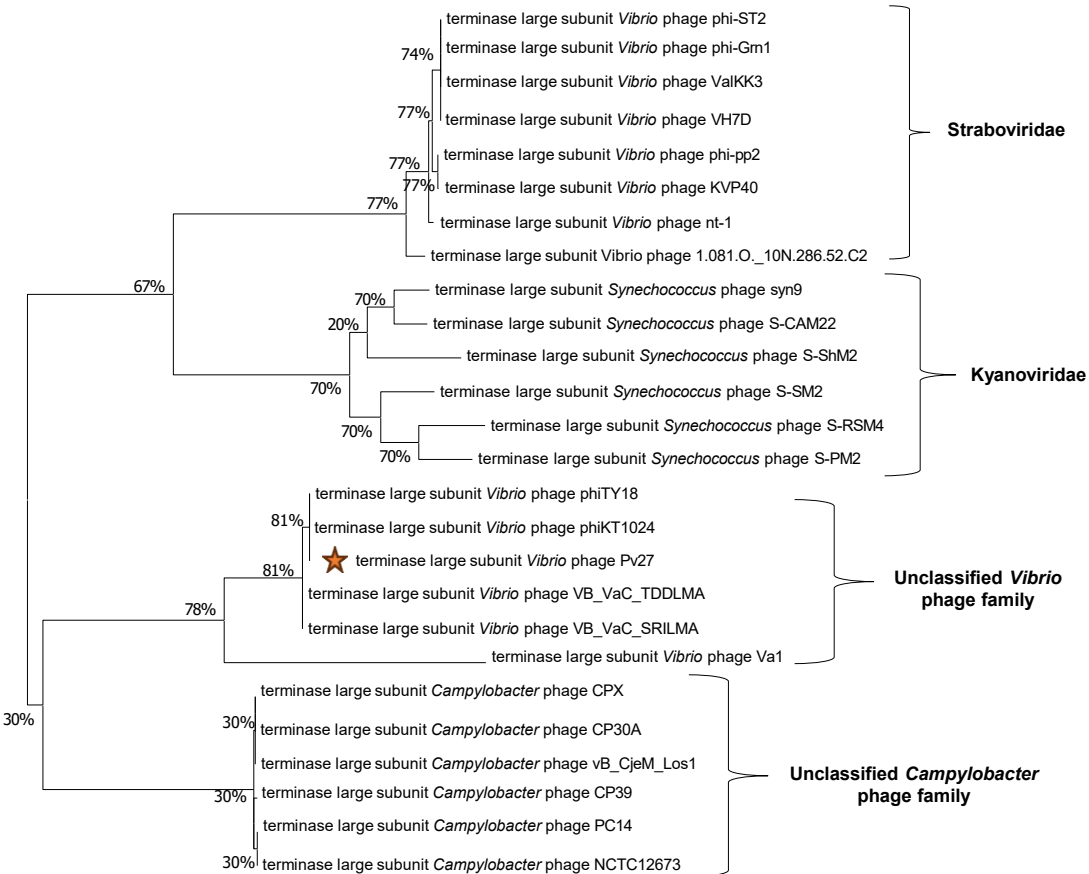

Supplement: Supplemental Information 4 — The phylogenetic tree was constructed using the Maximum Likehood with 1000 bootstrap replicates. Reference sequences were selected based on the top-hit BLAST results of phage Pv27’s terminase large subunit amino acid sequences. [file peerj-13-19421-s004.pdf]
